# Clinical features of Japanese patients with exacerbations of chronic obstructive pulmonary disease

**DOI:** 10.1186/s12890-020-01362-w

**Published:** 2020-12-07

**Authors:** Hiroki Tashiro, Yuki Kurihara, Koichiro Takahashi, Hironori Sadamatsu, Tetsuro Haraguchi, Ryo Tajiri, Ayako Takamori, Shinya Kimura, Naoko Sueoka-Aragane

**Affiliations:** 1 Division of Hematology, Respiratory Medicine and Oncology, Department of Internal Medicine, Faculty of Medicine, Saga University, 5-1-1 Nabeshima, Saga, Saga Prefecture 849-8501 Japan; 2 Clinical Research Center, Saga University Hospital, Saga, Japan

**Keywords:** Exacerbation, Frequency, Blood eosinophil

## Abstract

**Background:**

Exacerbations are critical events in chronic pulmonary obstructive disease (COPD). The frequency of COPD exacerbations is associated with the prognosis, including mortality, but no useful biomarker has been established.

**Methods:**

The present retrospective study investigated 481 COPD patients. Clinical features in the stable period were compared between patients who experienced severe exacerbation (n = 88, 18.3%) and those who never experienced severe exacerbation (n = 393, 81.7%). In the patients who experienced exacerbations, clinical features were also compared between frequent exacerbators (exacerbation rate ≥ 2 times/year, n = 27, 30.7%) and infrequent exacerbators (1 time/year, n = 61, 69.3%).

**Results:**

Compared to COPD patients who never experienced exacerbations, body mass index (BMI), serum albumin, and pulmonary functions were significantly lower, and the cardiovascular disease comorbidity rate, COPD assessment test score, modified Medical Research Council dyspnea scale, and use of long-term oxygen therapy, long-acting β_2_ adrenergic agonist therapy, inhaled corticosteroid therapy, and macrolide therapy were significantly higher in COPD patients with exacerbations (all p < 0.01). In patients who experienced exacerbations, frequent exacerbators had significantly lower % forced expiratory volume in 1.0 s and a higher risk of critical exacerbations, percentage of blood eosinophils, history of mechanical ventilation use, and use of long-term oxygen therapy and of macrolide therapy than infrequent exacerbators (all p < 0.01). On multivariate analysis, the percentage of blood eosinophils was the parameter most correlated with exacerbation frequency (β value [95% confidence interval] 1.45 [1.12–1.88], p < 0.01).

**Conclusion:**

Blood eosinophil in the stable period is the factor most correlated with the frequency of severe exacerbations.

*Trial registration*: The patients in this study was registered retrospectively

## Background

Chronic pulmonary obstructive disease (COPD) is a common respiratory disease, with a reported global prevalence of 251 million cases, and it is also an important life-threatening lung disease that is predicted to become the third leading cause of death worldwide by 2030 [1]. Exacerbation is one of the crucial events in patients with COPD, triggered by respiratory infections with bacteria and viruses and other factors such as pollution [2, 3]. It is also associated with worsening of mortality, lung function, and health-related quality of life. For example, a large real-world dataset of 177,207 patients with COPD in Japan showed that higher mortality is associated with pneumonia caused by microbes and aspiration pneumonia, which are causative factors for acute exacerbation of COPD [4]. Asian and Western studies also reported that exacerbation has a negative effect on COPD-associated quality of life (QOL), with decreased lung function [5–7]. The severity and frequency of exacerbations should be considered when attempting to predict the prognosis of COPD patients. In a prospective cohort of 304 men with COPD followed-up for 5 years, exacerbations requiring hospitalization showed an independent negative impact on patient prognosis [8]. Others have identified that moderate to severe exacerbation has a greater association with in-hospital death than mild exacerbation [9], and the 1-year mortality rate was also significantly higher for patients admitted to the intensive care unit for respiratory failure [10]. Additionally, frequent exacerbators showed a poor prognosis, with future frequent exacerbations identified as an independent risk factor in a 1-year prospective observational trial involving 90 Japanese patients with COPD [11]. To predict and prevent a worse clinical course in COPD patients, a more precise biomarker for frequent and severe exacerbations is necessary.

There is increasing evidence supporting the use of blood eosinophils as an accessible and beneficial biomarker for exacerbations in COPD patients. In particular, the percentage of blood eosinophils is considered a predictor of the effectiveness of inhaled corticosteroid (ICS) therapy to attenuate exacerbations [12, 13]. In patients with a percentage of blood eosinophils > 2%, ICS had a beneficial effect, reducing the exacerbation rate compared to those with a percentage of blood eosinophils < 2% [14]. Recently, the IMPACT study, which included moderate-to-very-severe COPD patients with at least one moderate or severe exacerbation in the previous year, also showed that ICS attenuated exacerbations for COPD patients with higher blood eosinophil counts [15]. Thus, the blood eosinophil has potential as a predictive biomarker of exacerbations in COPD patients, but it is still unclear how important it is for severe and frequent exacerbations in Japanese patients with COPD compared to other parameters such as airway obstruction, history of mechanical ventilation use, long-term oxygen therapy, and other treatments.

In this study, the initial focus was on characteristics of COPD patients, which were compared between patients who experienced severe exacerbations and those who never experienced severe exacerbations to clarify the factors related to severe exacerbations. Second, parameters associated with the frequency of severe exacerbations were identified, and multivariate analysis was performed to identify the parameters most related to exacerbation frequency. The percentage of blood eosinophils in the stable period was found to be the factor most correlated with the frequency of exacerbations, rather than FEV1.0% predicted, history of mechanical ventilation use, and long-term oxygen therapy. These results will contribute to the practical prediction of the prognosis of COPD patients with frequent severe exacerbations and to the selection of treatment including ICS.

## Methods

### Patients and setting

A total of 729 patients with the International Classification of Diseases, 10th Revision, Clinical Modification [ICD-10-CM] code for COPD (J449) in their medical records at the Saga University Hospital between 2009 and 2019 were retrospectively reviewed. From among these patients, two expert pulmonary physicians extracted 481 patients with COPD, considering the following definition criteria referring to the previous reports [16–19]: (1) age > 40 years; (2) forced expiratory volume in 1.0 s (FEV1.0)/forced vital capacity (FVC) ratio < 0.7; (3) smoking index > 10 pack-years; (4) persistent respiratory symptoms such as dyspnea, cough, and sputum; and (5) no medical history or current diagnosis of asthma. A severe exacerbation was defined as any sustained increase in respiratory symptoms compared with the stable phase and need for hospitalization considering symptoms, respiratory condition, and clinical findings with treatment of systemic corticosteroid or antibiotics, alone or combination. The need for hospitalization with an exacerbation was determined by the pulmonary physician. Causes of exacerbations were classified into 3 groups as bacterial infection, viral infection, and others, including unknown, considering respiratory sample cultures, examination of viral antigens for influenza, and radiological findings with physical findings. To standardize exacerbation frequency, the annual exacerbation rate was determined, and the maximum number of severe exacerbations per year from 2009 to 2019 was examined. Additionally, an infrequent exacerbator was defined as a patient whose maximum annual exacerbation rate was 1/year, and a frequent exacerbator was defined as a patient whose maximum annual exacerbation rate was more than 2/year. To assess the correlations between the maximum annual exacerbation rate and clinical factors, patients’ characteristics, COPD assessment test (CAT) score, modified Medical Research Council (mMRC) dyspnea scale, laboratory data, pulmonary function test results, transtricuspid pressure gradient (TRPG) on echocardiography, and treatment regimens were evaluated in the stable phase which was defined as no use of oral corticosteroids or antibiotics, no unscheduled doctor’s visit, or no hospitalization due to exacerbation of COPD in the past 4 weeks. Blood eosinophil was also evaluated in the stable phase according to the previous report [20]. For patients who never experienced exacerbations, the newest data in the medical record were evaluated. A critical exacerbation was defined as one with a fatal outcome. CO_2_ retention was defined as CO_2_ > 45 mmHg on arterial blood gas analysis. This study was approved by the ethics committee of Saga University Hospital (approval number: 2020-01-R-04, approval date: Mar 30, 2020) and was performed in accordance with the 1964 Declaration of Helsinki.

### Comorbidities

Hypertension, diabetes mellitus, hyperlipidemia and cardiovascular disease were diagnosed by the physicians. Cardiovascular disease included coronary artery disease, valvular disease, cardiac arrhythmias such as atrial fibrillation, and chronic heart failure diagnosed by echocardiography.

### Statistical analysis

The clinical data were analyzed by Student’s *t*-test for continuous variables or the chi-squared test for categorical variables. In patients who experienced exacerbations, univariate analysis was performed to extract significant variables in patients with infrequent exacerbations versus those with frequent exacerbations. To evaluate the most highly predictive variables in these 2 groups, multivariate analysis with logistic regression analysis was performed for categorical variables, and multiple linear regression analysis was performed for continuous variables. Quantitative data are presented as means ± standard deviation (SD), and odds ratios or β coefficient values were calculated. Statistical analysis was performed with JMP Pro version 14.2.0 software (SAS Institute Inc., Cary, NC, USA).

## Results

### Clinical characteristics of COPD patients with and without severe exacerbations

In the present study, 481 patients with COPD were analyzed. To examine the clinical impact of severe exacerbations, the 481 patients were divided into 393 patients who never experienced severe exacerbations and 88 patients who experienced severe exacerbations from 2009 to 2019. There were no significant differences in age, sex, height, and smoking history, but weight and the body mass index were significantly lower in patients with exacerbations than in those without exacerbations (p < 0.01, respectively). As comorbidities, hypertension, diabetes mellitus, and hyperlipidemia were not different in the 2 groups, but cardiovascular disease was significantly more common in patients with exacerbations than in those without exacerbations (30.7% vs 15.4%, p < 0.01). The CAT score and the mMRC dyspnea scale were higher in patients with exacerbations than in those without exacerbations (both p < 0.01). Of the laboratory data, the white blood cell count and blood eosinophil were not different between the 2 groups, but serum albumin was significantly lower in patients with exacerbations than in those without exacerbations (p < 0.01). On pulmonary function testing, VC, FVC, FEV 1.0, FEV1.0/FVC, %FEV1.0, and diffusing capacity of the lung for carbon monoxide (DLco) were significantly lower in patients with severe exacerbations than in those without severe exacerbations (all p < 0.01). TRPG, which is one of the predictors of pulmonary hypertension, was also higher in patients with exacerbations (p < 0.01). As for treatment, long-term oxygen therapy, long acting β_2_ adrenergic agonists (LABAs), ICS, and macrolides were used significantly more in patients with exacerbations than in those without exacerbations (all p < 0.01) (Table 1). These data showed that severe exacerbations were closely associated with the physical and clinical characteristics of patients with COPD.

**Table 1:** Clinical characteristics in COPD patients with severe exacerbation versus those without severe exacerbation

	Non exacerbator	Exacerbator	p value
n	393	88	
Age (years)	71.6 ± 0.4	73.3 ± 1.0	0.1
Gender (male/female)	371/22	83/5	0.98
Height	162.9 ± 0.4	162.1 ± 0.8	0.62
Body weight	59.1 ± 0.6	53.5 ± 1.1	< 0.01
BMI (kg/m^2^)	22.1 ± 0.2	20.3 ± 0.4	< 0.01
Smoking history (pack-year)	58.9 ± 1.6	63.0 ± 2.7	0.29
Comorbidity			
Hypertension	185 (47.1%)	42 (47.7%)	0.91
Diabetes mellitus	77 (19.7%)	18 (20.5%)	0.87
Hyperlipidemia	60 (15.4%)	15 (17.1%)	0.69
Cardiovascular disease	60 (15.4%)	27 (30.7%)	< 0.01
COPD assessment test	10.3 ± 0.5	19.9 ± 1.3	< 0.01
mMRC dyspnea scale, grades	1.48 ± 0.12	2.67 ± 0.71	< 0.01
Laboratory data			
White blood cell (/ml)	6652.4 ± 82.4	6830.7 ± 194.6	0.36
Blood eosinophil (%)	2.73 ± 0.11	2.84 ± 0.27	0.66
Blood eosinophil count (/ml)	175.4 ± 7.4	184.2 ± 17.4	0.61
Serum albumin (g/dl)	3.81 ± 0.02	3.60 ± 0.06	< 0.01
Pulmonary function test			
VC (L)	3.16 ± 0.04	2.84 ± 0.09	< 0.01
FVC (L)	3.02 ± 0.04	2.65 ± 0.09	< 0.01
FEV1.0 (L)	1.66 ± 0.03	1.24 ± 0.06	< 0.01
FEV1.0/FVC (%)	54.4 ± 0.0	46.6 ± 0.0	< 0.01
%FEV1.0 (%)	75.4 ± 1.1	58.6 ± 2.6	< 0.01
DLco (%)	71.0 ± 2.3	55.7 ± 3.7	< 0.01
TRPG (mmHg)	27 ± 1.1	38.5 ± 2.2	< 0.01
Treatment			
Long-term oxygen therapy	20 (5.1%)	42 (47.7%)	< 0.01
LAMA	176 (45.1%)	49 (55.7%)	0.07
LABA	155 (39.7%)	60 (68.2%)	< 0.01
ICS	73 (18.7%)	41 (46.6%)	< 0.01
Macrolide	27 (7.0%)	23 (26.1%)	< 0.01
β blocker	37 (9.5%)	12 (13.6%)	0.27

*COPD* chronic obstructive pulmonary disease, *BMI* body mass index, *mMRC* modified medical research council, *VC* vital capacity, *FVC* forced vital capacity, *FEV1.0* forced expiratory volume in 1.0 s, *DLco* diffusing capacity of lung for carbon monoxide, *TRPG* transtricuspid pressure gradient, *LAMA* long acting muscarinic antagonist, *LABA* long acting β_2_ adrenergic agonist, *ICS* inhaled corticosteroid. Data are presented as mean ± standard deviation

### Evaluation of the frequency and causes of exacerbations in patients with COPD

To clarify the impact of exacerbation frequency in patients with COPD, the analysis focused on the 88 patients who experienced exacerbations, and the annual exacerbation rate was extracted for the maximum number of severe exacerbations in 1 year from 2009 to 2019. The 88 patients were divided into those whose annual exacerbation rate was once/year (infrequent exacerbators, 62 patients) and the 26 patients whose annual exacerbation rate was ≥ 2 times/year (frequent exacerbators). The annual exacerbation rate of the frequent exacerbators was 2.6 ± 0.3 (range 2–8 times). The total number of exacerbations from 2009 to 2019 was 65 in infrequent exacerbators and 111 in frequent exacerbators. As the causes of the exacerbations, bacterial infection was the most common, and viral infection and others were uncommon in the present study (Table 2).

**Table 2:** Frequency and causes for exacerbation in patients with COPD

	Infrequent exacerbator	Frequent exacerbator
n	61	27
Annual exacerbation rate	1	2.6 ± 0.3
Total events of exacerbation	65	111
Causes of exacerbations		
Bacterial infection	47 (72.3%)	91 (82%)
Viral infection	3 (4.6%)	4 (3.6%)
Others include unknown	15(23.1%)	16 (14.4%)

Data are presented as mean ± standard deviation

### Comparison of clinical characteristics in COPD patients with and without frequent exacerbations

To address the clinical impact of exacerbation frequency, clinical characteristics were compared between infrequent exacerbators and frequent exacerbators. BMI was significantly lower in frequent exacerbators than in infrequent exacerbators (p = 0.03). Critical exacerbations with fatal outcomes were significantly more common in frequent exacerbators (p < 0.01). There were no differences in comorbidities and the CAT score in these 2 groups. As for the laboratory data, the percentage of blood eosinophils and the blood eosinophil count were significantly higher in frequent exacerbators than in infrequent exacerbators (p < 0.01, p = 0.03 respectively). The frequency of patients with CO_2_ retention (> 45 mmHg) on arterial blood gas analysis was higher in frequent exacerbators (p = 0.05). On pulmonary function test results, %FEV1.0 was significantly lower in frequent exacerbators than in infrequent exacerbators (p < 0.01). TRPG was not different between the 2 groups, and a history of mechanical ventilation use for exacerbations was significantly higher in frequent exacerbators (p < 0.01). As for treatment, the frequencies of the use of long-term oxygen therapy, long-acting muscarinic antagonists (LAMAs), and macrolides was higher in frequent exacerbators than in infrequent exacerbators (p < 0.01, p = 0.02, p < 0.01 respectively), but the use of ICS was not different between the 2 groups (Table 3). We also compared clinical characteristics between COPD patients with frequent exacerbations and those without exacerbations. BMI and pulmonary function testing including VC, FVC, FEV 1.0, FEV1.0/FVC, %FEV1.0 are significantly lower (all p < 0.01) and CAT score, blood eosinophil, TRPG are significantly higher in patients with frequent exacerbations than in those without exacerbations (p < 0.01, p = 0.02, p < 0.01 respectively). As for treatment, long-term oxygen therapy, LAMA, LABA, ICS, and macrolides were used significantly more in patients with frequent exacerbations than in those without exacerbations (all p < 0.01) (Additional file 1: Table E1).

**Table 3:** Comparison of clinical characteristics in COPD patients with frequent exacerbation versus those with infrequent exacerbation

	Infrequent exacerbator	Frequent exacerbator	Univariate analysis
n	61	27	
Age (years)	74.0 ± 1.2	71.7 ± 0.4	0.3
BMI (kg/m^2^)	20.9 ± 0.4	19.0 ± 0.8	0.03
Smoking history (pack-year)	61.2 ± 4.4	67.3 ± 7.5	0.46
Comorbidity			
Hypertension	30 (49.2%)	12 (44.4%)	0.68
Diabetes mellitus	10 (16.4%)	8 (29.6%)	0.17
Hyperlipidemia	12 (19.7%)	3 (11.1%)	0.31
Cardiovascular disease	19 (31.2%)	8 (30.7%)	0.89
COPD assessment test	19.8 ± 2.1	20.0 ± 1.7	0.94
Critical exacerbation	4 (6.6%)	10 (38.5%)	< 0.01
Laboratory data			
White blood cell (/ml)	7067.2 ± 217.8	6296.3 ± 387.1	0.07
Blood eosinophil (%)	2.44 ± 0.29	3.76 ± 0.55	< 0.01
Blood eosinophil count (/ml)	154.7 ± 16.9	233.4 ± 37.4	0.03
Serum albumin (g/dl)	3.60 ± 0.07	3.62 ± 0.09	0.84
CO_2_ retention	12/60(20%)	11/27 (40.7%)	0.05
Pulmonary function test			
VC (L)	2.89 ± 0.11	2.73 ± 0.14	0.42
FVC (L)	2.70 ± 0.11	2.52 ± 0.13	0.35
FEV1.0 (L)	1.32 ± 0.08	1.05 ± 0.09	0.04
FEV1.0/FVC (%)	48.6 ± 1.8	42.2 ± 2.6	0.05
%FEV1.0 (%)	63.3 ± 3.3	48.0 ± 3.5	< 0.01
DLco (%)	54.4 ± 4.6	59.3 ± 6.3	0.56
TRPG (mmHg)	36.2 ± 2.4	42.6 ± 4.5	0.18
History			
Mechanical ventilation use	7 (11.5%)	11 (40.7%)	< 0.01
Treatment			
Long-term oxygen therapy	21 (34.4%)	21 (77.8%)	< 0.01
Home ventilation use	4 (6.6%)	4 (14.8%)	0.23
LAMA	29 (47.5%)	20 (74.1%)	0.02
LABA	38 (62.3%)	22 (81.5%)	0.07
ICS	29 (47.5%)	12 (44.4%)	0.79
Macrolide	10 (16.4%)	13 (48.1%)	< 0.01
β blocker	8 (13.1%)	4 (14.8%)	0.8

*COPD* chronic obstructive pulmonary disease, *BMI* body mass index, *VC* vital capacity, *FVC* forced vital capacity, *FEV1.0* forced expiratory volume in 1.0 s, *DLco* diffusing capacity of lung for carbon monoxide, *TRPG* transtricuspid pressure gradient, *LAMA* long acting muscarinic antagonist, *LABA* long acting β_2_ adrenergic agonist, *ICS* inhaled corticosteroid. Data are presented as mean ± standard deviation

### Multivariate analysis and correlation analysis of the clinical characteristics in COPD patients with frequent exacerbations versus those with infrequent exacerbations

To evaluate the most useful predictor of the frequency of exacerbations in COPD patients, multivariate analysis was performed using variables that were significant in frequent exacerbators compared to infrequent exacerbators, such as the rate of critical exacerbations, percentage of blood eosinophils, %FEV1.0, history of mechanical ventilation use, long-term oxygen therapy, macrolide use, BMI and rate of CO_2_ retentions on univariate analysis. Critical exacerbation (odds ratio or β value [95% confidence interval] 6.07 [1.06–36.67], p = 0.04) and mechanical ventilation use (5.49 [1.19–25.19], p = 0.03) were significant as independent predictors, and the percentage of blood eosinophils was the most significant of these variables (1.45 [1.12–1.88], p < 0.01) (Table 4). We also performed multivariate analysis using variables that were significant in patient with frequent exacerbations and in those without exacerbation. Long-term oxygen therapy (37.09 [3.95–348], p < 0.01) and the percentage of blood eosinophil (1.64 [1.12–2.64], p = 0.02) were significant as independent predictors (Additional file 1: Table E2).

**Table 4:** Multivariate analysis of the clinical characteristics in COPD patients with frequent exacerbation versus those with infrequent exacerbation

	Multivariate analysis		
	Adjusted OR or β	95% CI	p value
Critical exacerbation	6.07	1.06–34.67	0.04
Blood eosinophil (%)	1.45	1.12–1.88	< 0.01
%FEV1.0 (%)	0.3	0.01–7.01	0.46
Mechanical ventilation use	5.49	1.19–25.19	0.03
Long-term oxygen therapy	1.69	0.41–6.96	0.47
Macrolide	2.28	0.59–8.82	0.23
BMI	0.91	0.75–1.12	0.38
CO_2_ retention	1.16	0.23–5.83	0.85

*OR* odds ratio, *β* standardized β value, *CI* confidence interval, *BMI* body mass index

## Discussion

In the present real-world study, BMI, serum albumin, and pulmonary functions including VC, FVC, FEV1.0, FEV1.0/FVC, %FEV1.0, and DLco were significantly lower, and the cardiovascular disease comorbidity rate, COPD assessment test score, mMRC dyspnea scale, and use of long-term oxygen therapy, LABAs, ICS, and macrolides were significantly higher in COPD patients who experienced exacerbations than in COPD patients who never experienced severe exacerbations. Of patients who experienced exacerbations, frequent exacerbators had significantly lower BMI, FEV1.0, and %FEV1.0, and higher risk of critical exacerbations, blood eosinophils, history of mechanical ventilation use, use of long-term oxygen therapy, LAMAs, and macrolides than infrequent exacerbators. Multivariate analysis showed that the parameter most correlated with exacerbation frequency was the percentage of blood eosinophils.

Exacerbation is one of the independent phenotypes in COPD patients who develop worse health conditions, including symptoms and pulmonary function, which is supported by many previous reports [21, 22]. For example, there was an 8% increase in the risk of exacerbations per unit increase in CAT [23], and additional exacerbation was associated with a greater decline of 6–7 ml in FEV1.0 in a huge cohort study [24, 25], consistent with the results of the current study (Table 1). As for comorbidities, cardiovascular diseases were more common in patients who had exacerbations with significantly higher pulmonary artery pressure (Table 1). Cardiovascular events are common in COPD patients, and 20.5% of stable COPD patients had unrecognized heart failure [26, 27]; they were also significantly associated with long-term mortality in COPD patients with exacerbations [28].

Exacerbation frequency is also an important factor related to the prognosis of COPD patients. Donaldson et al. analyzed 109 patients with COPD who experienced 757 exacerbations and suggested that FEV1.0 was decreased at 40.1 ml/year in frequent exacerbators compared to 32.1 ml/year in infrequent exacerbators [25]. In a 1-year prospective observational trial of Japanese COPD patients, Tomioka et al. reported that frequent exacerbators had lower BMIs and higher CAT scores than infrequent exacerbators [11]. Moreover, the retrospective UPLIFT study of 6000 patients with COPD and the 3-year observational ECLIPS study of 2000 patients with COPD reported that the patients with higher exacerbation rates had more rapid lung function decline [29, 30]. Interestingly, more than 20% of COPD patients with GOLD stage 2 (50% < FEV1.0 < 80% predicted) had two or more annual exacerbations, and the frequency in the previous year predicted the future frequency [30]. These data suggest that frequency is also an independent phenotype of COPD patients with exacerbation.

The present results suggest that the percentage of blood eosinophils in the stable period is the factor most correlated with exacerbation frequency compared to other variables, including the critical exacerbation rate, %FEV1.0, history of mechanical ventilation use, use of long-term oxygen therapy, use of macrolides, BMI and CO_2_ retention rate (Table 4). A previous paper reported that COPD patients with a blood eosinophil count > 500 cells/μl had a 1.43 times higher risk of exacerbation in a huge database analysis [31], and others reported that patients with a blood eosinophil count > 340 cells/μl also had a 1.76-fold increased risk of severe exacerbation [32], which is consistent with the present result. Notably, the blood eosinophil was not significantly different between COPD patients who experienced exacerbations and those who never experienced exacerbations in the present study, which supported the association of blood eosinophils with exacerbation frequency, but not exacerbation itself (Table 1).

To decrease frequent exacerbations, ICS treatment was considered for suppression of airway inflammation. Recently, two randomized trials showed that ICS therapy suppressed moderate to severe exacerbations in COPD patients with improvement of pulmonary function [33, 34]. In that study, the COPD patients with blood eosinophil counts ≥ 310 cells/μl showed a greater reduction of the exacerbation rate with improvement of FEV1.0, symptoms, and QOL score than those with blood eosinophil counts < 90 cells/μl [15]. Importantly, the present results showed that the rate of patients treated by ICS was no different between frequent exacerbators and infrequent exacerbators (Table 3), which might contribute to the current prognostic results.

There are several limitations in this study. First, it was not possible to completely exclude patients with asthma-COPD overlap from the current study population. Because such patients had frequent exacerbations with higher blood eosinophil levels, the current results in frequent exacerbators might be affected by this population. Second, unreported exacerbations were also reported to be important for health status, as much as reported exacerbations, in COPD patients [35]. The effect of unreported exacerbations, which might have impacted the current results, was not evaluated. Third, the disease duration of COPD might be different between COPD patients with exacerbations and those without exacerbations which might influence of the present results. Fourth, decision of hospitalization for definition of severe exacerbation is depending on physician’s judgment which might also involve the present results. Finally, the present study involved patients at a single hospital with limited ethnic diversity and small sample size. To confirm the validity of the present results, multicenter prospective studies with a larger number of patients should be performed.

## Conclusions

The percentage of blood eosinophils in the stable period was the factor most correlated with the frequency of severe exacerbations, rather than FEV1.0%predicted, history of mechanical ventilation use, and long-term oxygen therapy. These results will contribute to predicting the prognosis of COPD patients and the selection of treatment including ICS.

## Supplementary information

**Additional file 1.** **Table E1.** Comparison of clinical characteristics in COPD patients with frequent exacerbation vs those without exacerbation. **Table E2.** Multivariate analysis of the clinical characteristics in COPD patients with frequent exacerbation vs those without exacerbation.

## Data Availability

The datasets used and analyzed during the current study are available from the corresponding author on reasonable request.

## Abbreviations

COPD: Chronic pulmonary obstructive disease
BMI: Body mass index
QOL: Quality of life
ICS: Inhaled corticosteroid
FEV1.0: Forced expiratory volume in 1.0 s
FVC: Forced vital capacity
CAT: COPD assessment test
mMRC: Modified Medical Research Council
TRPG: Transtricuspid pressure gradient
SD: Standard deviation
DLco: Diffusing capacity of the lung for carbon monoxide
LABAs: Long acting β_2_ adrenergic agonists
LAMAs: Long-acting muscarinic antagonists
OR: Odds ratio
β: Standardized β value
CI: Confidence interval
