# Supplementary material for: Clinical features of Japanese patients with exacerbations of chronic obstructive pulmonary disease
Source: BMC Pulm Med. 2020 Dec 7;20:318. doi: 10.1186/s12890-020-01362-w (PMC7720558; doi:10.1186/s12890-020-01362-w)
Supplement: Supplementary file 1 — Additional file 1. Table E1. Comparison of clinical characteristics in COPD patients with frequent exacerbation vs those without exacerbation. Table E2. Multivariate analysis of the clinical characteristics in COPD patients with frequent exacerbation vs those without exacerbation. [file 12890_2020_1362_MOESM1_ESM.docx]

**Online Data Supplement**

**The title**

Clinical features of Japanese patients with exacerbations of chronic obstructive pulmonary disease

**Authors**

Hiroki Tashiro, Yuki Kurihara, Koichiro Takahashi, Hironori Sadamatsu, Tetsuro Haraguchi, Ryo Tajiri, Ayako Takamori, Shinya Kimura, Naoko Sueoka-Aragane

**Table E1 Comparison of clinical characteristics in COPD patients with frequent exacerbation vs those without exacerbation**

|  | **Non exacerbator** | **Frequent exacerbator** | **Univariate analysis** |
| --- | --- | --- | --- |
| **n** | 393 | 27 |  |
| **Age (years)** | 71.6 ± 0.4 | 71.7 ± 0.4 | 0.94 |
| **BMI (Kg/m2)** | 22.1 ± 0.2 | 19.0 ± 0.8 | <0.01 |
| **Smoking history (pack-year)** | 58.9 ± 1.6 | 67.3 ± 7.5 | 0.19 |
|  |  |  |  |
| **Comorbidity** |  |  |  |
| **Hypertension** | 185 (47.1%) | 12 (44.4%) | 0.8 |
| **Diabetes mellitus** | 77 (19.7%) | 8 (29.6%) | 0.24 |
| **Hyperlipidemia** | 60 (15.4%) | 3 (11.1%) | 0.54 |
| **Cardiovascular disease** | 60 (15.4%) | 8 (30.7%) | 0.07 |
|  |  |  |  |
| **COPD assessment test** | 10.3 ± 0.5 | 20.0 ± 1.7 | <0.01 |
|  |  |  |  |
| **Laboratory data** |  |  |  |
| **White blood cell (/ml)** | 6652.4 ± 82.4 | 6296.3 ± 387.1 | 0.27 |
| **Blood eosinophil (%)** | 2.73 ± 0.11 | 3.76 ± 0.55 | 0.02 |
| **Blood eosinophil count (/ml)** | 175.4 ± 7.4 | 233.4 ± 37.4 | 0.05 |
| **Serum albumin (g/dl)** | 3.81 ± 0.02 | 3.62 ± 0.09 | 0.04 |
|  |  |  |  |
| **Pulmonary function test** |  |  |  |
| **VC (L)** | 3.16 ± 0.04 | 2.73 ± 0.14 | <0.01 |
| **FVC (L)** | 3.02 ± 0.04 | 2.52 ±0.13 | <0.01 |
| **FEV1.0 (L)** | 1.66 ± 0.03 | 1.05 ± 0.09 | <0.01 |
| **FEV1.0/FVC (%)** | 54.4 ± 0.0 | 42.2 ± 2.6 | <0.01 |
| **%FEV1.0 (%)** | 75.4 ± 1.1 | 48.0 ± 3.5 | <0.01 |
| **DLco (%)** | 71.0 ± 2.3 | 59.3 ± 6.3 | 0.13 |
|  |  |  |  |
| **TRPG (mmHg)** | 27 ± 1.1 | 42.6 ± 4.5 | <0.01 |
|  |  |  |  |
| **Long-term oxygen therapy** | 20 (5.1%) | 21 (77.8%) | <0.01 |
| **LAMA** | 176 (45.1%) | 20 (74.1%) | <0.01 |
| **LABA** | 155 (39.7%) | 22 (81.5%) | <0.01 |
| **ICS** | 73 (18.7%) | 12 (44.4%) | <0.01 |
| **Macrolide** | 27 (7.0%) | 13 (48.1%) | <0.01 |
| **β blocker** | 37 (9.5%) | 4 (14.8%) | 0.4 |

COPD; chronic obstructive pulmonary disease, BMI; body mass index, VC; vital capacity, FVC; forced vital capacity, FEV1.0; forced expiratory volume in 1.0 second, DLco; diffusing capacity of lung for carbon monoxide; TRPG; transtricuspid pressure gradient, LAMA; long acting muscarinic antagonist, LABA; long acting β_2_ adrenergic agonist,

ICS; inhaled corticosteroid. Data are presented as mean ± standard deviation.

**The title**

Clinical features of Japanese patients with exacerbations of chronic obstructive pulmonary disease

**Authors**

Hiroki Tashiro, Yuki Kurihara, Koichiro Takahashi, Hironori Sadamatsu, Tetsuro Haraguchi, Ryo Tajiri, Ayako Takamori, Shinya Kimura, Naoko Sueoka-Aragane

**Table E2 Multivariate analysis of the clinical characteristics in COPD patients with frequent exacerbation vs those without exacerbation**

|  | **Multivariate analysis** | | |
| --- | --- | --- | --- |
|  | **Adjusted OR or β** | **95% CI** | **p value** |
| **BMI** | 0.96 | 0.65-1.38 | 0.83 |
| **COPD assessment test** | 1 | 0.87-1.16 | 0.98 |
| **Blood eosinophil (%)** | 1.64 | 1.12-2.64 | 0.02 |
| **Serum albumin (g/dl)** | 0.14 | 0.01-1.29 | 0.08 |
| **%FEV1.0 (%)** | 0.01 | 0.0000061-6.38 | 0.2 |
| **Long-term oxygen therapy** | 37.09 | 3.95-348 | <0.01 |
| **LAMA** | 2.06 | 0.1-40.94 | 0.64 |
| **Macrolide** | 2.39 | 0.24-24.05 | 0.46 |

OR; odds ratio, β; standardized β value, CI; Confidence interval, BMI; body mass index, FEV1.0; forced expiratory volume in 1.0 second
